# HIPK4 accelerates cutaneous squamous cell carcinoma progression by phosphorylating TAp63 and inhibiting EFEMP1 expression

**DOI:** 10.1016/j.jbc.2025.108564

**Published:** 2025-04-30

**Authors:** Ze Guo, Bingjie Chen, Mengya Zhang, Min Gao, Zaixing Wang, Huayang Tang, Xianfa Tang, Qian Zhang, Jochen Utikal

**Affiliations:** 1 Department of Dermatology, the First Affiliated Hospital of Anhui Medical University, Hefei, Anhui Province, P.R. China; 2 Department of Dermatology, Nanjing Hospital of Chinese Medicine Affiliated to Nanjing University of Chinese Medicine, Nanjing, Jiangsu Province, P.R. China; 3 Skin Cancer Unit, German Cancer Research Center (DKFZ), Heidelberg, Germany; 4 Department of Dermatology, Venereology and Allergology, University Medical Center Mannheim, Ruprecht-Karl University of Heidelberg, Mannheim, Germany; 5 DKFZ Hector Cancer Institute at the University Medical Center Mannheim, Mannheim, Germany

**Keywords:** cutaneous squamous cell carcinoma, EFEMP1, HIPK4, TAp63, phoshorylation

## Abstract

Cutaneous squamous cell carcinoma (CSCC) is a common skin cancer with a tendency to metastasize, leading to poor patient prognosis. Homeodomain interacting protein kinase 4 (HIPK4) has been identified as a key inhibitor of human skin epithelial differentiation. However, the role of HIPK4 in regulating CSCC development remains unclear. Our preliminary experiment showed that HIPK4 was highly expressed in CSCC tumor tissues and cells. In this study, we investigate the role of HIPK4 in regulating CSCC progression and the underlying mechanisms. In the current study, the interaction between HIPK4 and TAp63 was analyzed by Co-IP and GST-pull down assays, and the relationship between TAp63 and *EFEMP1* was analyzed by ChIP and dual luciferase reporter assays. Our results showed that EFEMP1 expression was decreased in CSCC tissues and cells, and EFEMP1 overexpression inhibited CSCC cell proliferation, migration, and invasion. In addition, HIPK4 was upregulated in CSCC; knocking down HIPK4 suppressed CSCC cell malignant behaviors and tumor growth in mice. Mechanistically, HIPK4 promoted tumor progression by phosphorylating the tumor suppressor TAp63 at Ser395, leading to decreased expression of EFEMP1, a key extracellular matrix protein with anti-tumor properties. As expected, the inhibitory effects of HIPK4 knockdown on CSCC cell malignant behaviors were reversed by EFEMP1 knockdown. In summary, HIPK4 could exacerbate CSCC malignant progression by inhibiting EFEMP1 through phosphorylating TAp63, highlighting HIPK4 as a potential therapeutic target in CSCC. Our results provide new insights into the molecular mechanisms underlying CSCC progression and propose novel strategies for therapeutic intervention.

Cutaneous squamous cell carcinoma (CSCC) is the second most prevalent skin cancer, and its incidence is rising (1). Despite recent breakthroughs in clinical treatment of CSCC, including surgery, immunotherapy, EGFR-targeted therapy, and radiotherapy, the overall survival of patients with CSCC is not ideal (2). Notably, the 5-year disease-specific survival of individuals with CSCC was 95.7%, with a mean survival time of 18.6 years (3). As a result, there is an urgent need to find innovative treatment techniques to inhibit the malignant progression of CSCC, and understanding the molecular mechanisms involved is key to achieving this goal.

The p63 is a transcription factor that is essential for the development and maintenance of squamous epithelial cells (4). The *p63* gene encodes two major protein isoforms, TAp63 and ΔNp63 (each containing TA or ΔN domain) (5). TAp63 contains a shared trans-activation domain similar to p53, which can simulate the p53 function, while ΔNp63 lacks this domain and acts as a dominant negative to p53 (4). TAp63 is a tumor-suppressor protein that is often expressed lowly in multiple human malignant tumors (6, 7). According to the transcriptional domain, TAp63 can activate gene transcription and induce cell cycle arrest and apoptosis (8). Based on these features, TAp63 has been shown to be a key player in restraining metastatic cancer development (9). The role of TAp63 in CSCC has also been studied. As evidence, TAp63 overexpression inhibited the progression and metastasis of mouse and human CSCC (10). Moreover, TAp63^−/−^ mice presented an increased susceptibility to ultraviolet radiation-induced CSCC (11). TAp63 is a tumor and metastasis suppressor in CSCC, while its mechanism of action remains unknown.

Homeodomain interacting protein kinase 4 (HIPK4) is a member of the HIPKs family, which is a type of serine/threonine kinase (12). HIPK4 is identified as an essential regulator in the development of human skin epithelial cells and keratinocyte generation (13). However, the effect of HIPK4 on CSCC remains unclear and deserves further research. As a conserved serine/threonine kinase, the main mechanism by which HIPK4 functions is through phosphorylation of specific proteins (14). A previous study showed that HIPK4 could phosphorylate human p53 protein at serine nine and inhibit p53-mediated transcriptional regulation (15). Given the highly similar structure of TAP63 and p53, we speculated that HIPK4 can also phosphorylate the TAP63 protein. Using the String database (https://string-db.org/), the current study hypothesized that HIPK4 may interact with the TAp63 protein. In addition, combined with Uniprot (https://www.uniprot.org/) and phosphosite (http://www.phosphosite.org) analysis, we found a possible phosphorylation site between HIPK4 and p63, located at Ser395. Therefore, we supposed that HIPK4 may accelerate the malignant progression of CSCC by phosphorylating TAp63 at Ser395 and inhibiting its transcriptional activity.

Using the JASPAR database (http://jaspar.genereg.net/), we predicted that p63 had potential binding sites to *EFEMP1*. EGF-containing fibulin-like extracellular matrix protein 1 (EFEMP1) is a member of the fibulin family of extracellular matrix glycoproteins and is crucial in maintaining extracellular matrix structure stability (16). Previous research has found that EFEMP1 is connected with the tumorigenicity of certain malignancies. As proof, Song *et al.* revealed that EFEMP1 upregulation promoted cervical cancer cell proliferation, invasion, and adhesion (17). Furthermore, similar findings were seen in investigations of pancreatic cancer and glioma (18, 19). In contrast to the abovementioned findings, reports on CSCC showed that EFEMP1 was reduced in CSCC tissues, and EFEMP1 upregulation reduced CSCC cell proliferation, migration, and invasion (20). EFEMP1 presents anti-tumorigenic activities in CSCC. Nevertheless, the interaction between TAp63 and EFEMP1 in regulating the malignant progression of CSCC is still unknown and deserves further investigation.

Based on the abovementioned research, we presumed that HIPK4 could phosphorylateTAp63 and inhibit EFEMP1, thereby facilitating CSCC malignant development. Our study might provide a theoretical basis for the development of novel treatment strategies for CSCC.

## Results

### HIPK4 expression and TAp63 phosphorylation were markedly increased in CSCC, and EFEMP1 was downregulated

**Figure 1:**
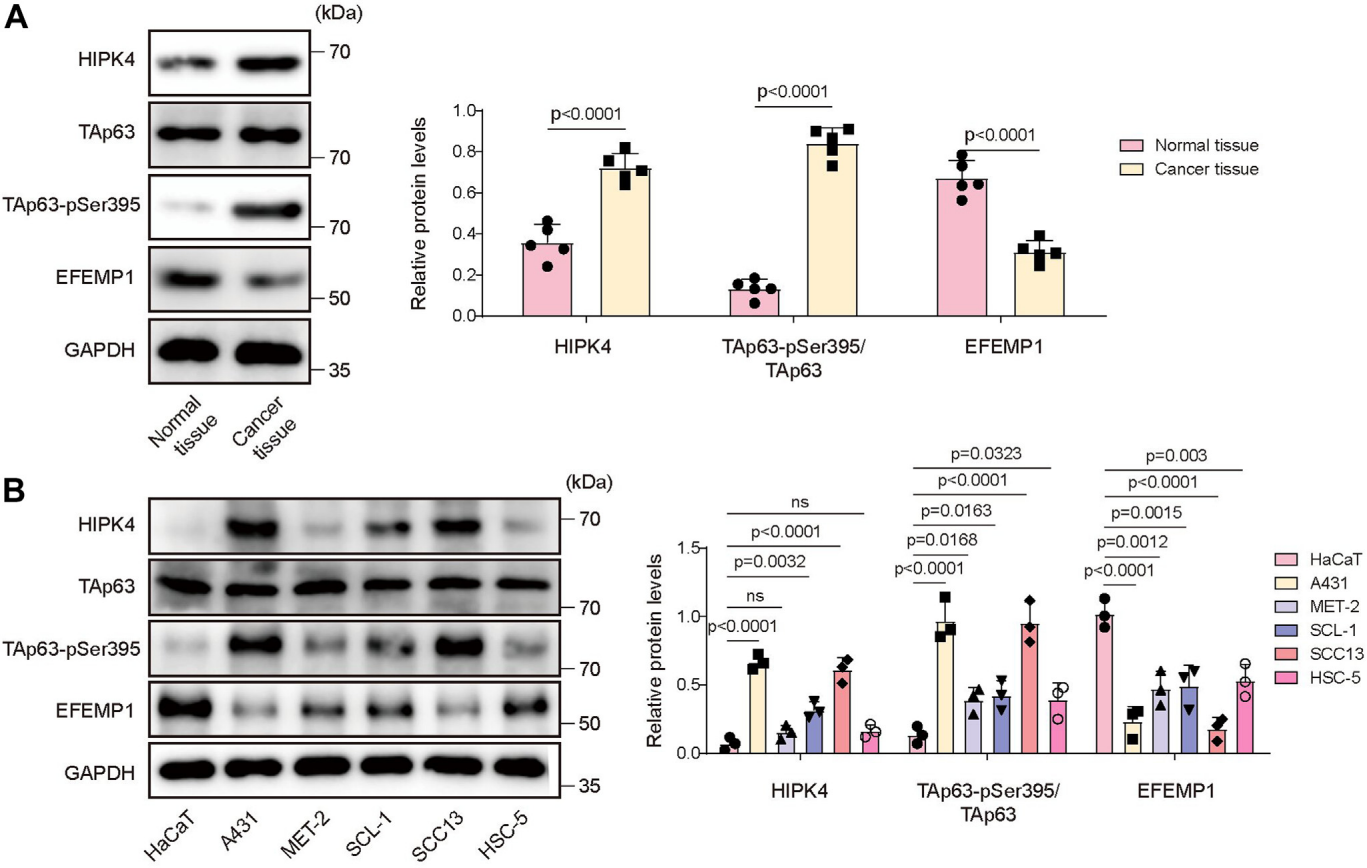
**HIPK4 expression and TAp63 phosphorylation were markedly increased in CSCC, and EFEMP1 was downregulated.***A*, CSCC tumor tissues and paired adjacent normal tissues were collected from patients with CSCC, and the protein levels of HIPK4, TAp63, TAp63-pSer395, and EFEMP1 in tissues were detected by Western blot (n = 5); *p* < 0.01 for HIPK4, *p* < 0.001 for TAp63-pSer395/TAp63, *p* < 0.01 for EFEMP1. *B*, Western blot was employed to determine HIPK4, TAp63, TAp63-pSer395, and EFEMP1 protein levels in human immortalized epidermal cells (HaCaT cells) and human CSCC cell lines (A431, MET-2, SCL-1, SCC13, and HSC-5 cells); *p* < 0.01 for HIPK4, *p* < 0.05 for TAp63-pSer395/TAp63, *p* < 0.01 for EFEMP1. The differences between the two groups were investigated using Student’s t-tests. One-way ANOVA was performed to compare differences between groups. All data were obtained from at least three replicate experiments and was presented as mean ± standard deviation (SD).

CSCC tumor tissues and adjacent tissues were collected from patients with CSCC, and we observed that HIPK4 and TAp63-pSer395 levels were significantly increased in CSCC tumor tissues compared with normal tissues, while EFEMP1 expression was reduced (n = 5) (Fig. 1*A*). Meanwhile, HIPK4 and TAp63-pSer395 levels were elevated in CSCC cell lines (A431, MET-2, SCL-1, SCC13, and HSC-5 cells) compared with those in human immortalized epidermal cells (HaCaT cells); however, the EFEMP1 protein was lowly expressed in CSCC cell lines (Fig. 1*B*). Collectively, the dysregulation of HIPK4, TAp63 phosphorylation, and EFEMP1 might be related to CSCC progression.

### EFEMP1 overexpression inhibited CSCC cell malignant behaviors

**Figure 2:**
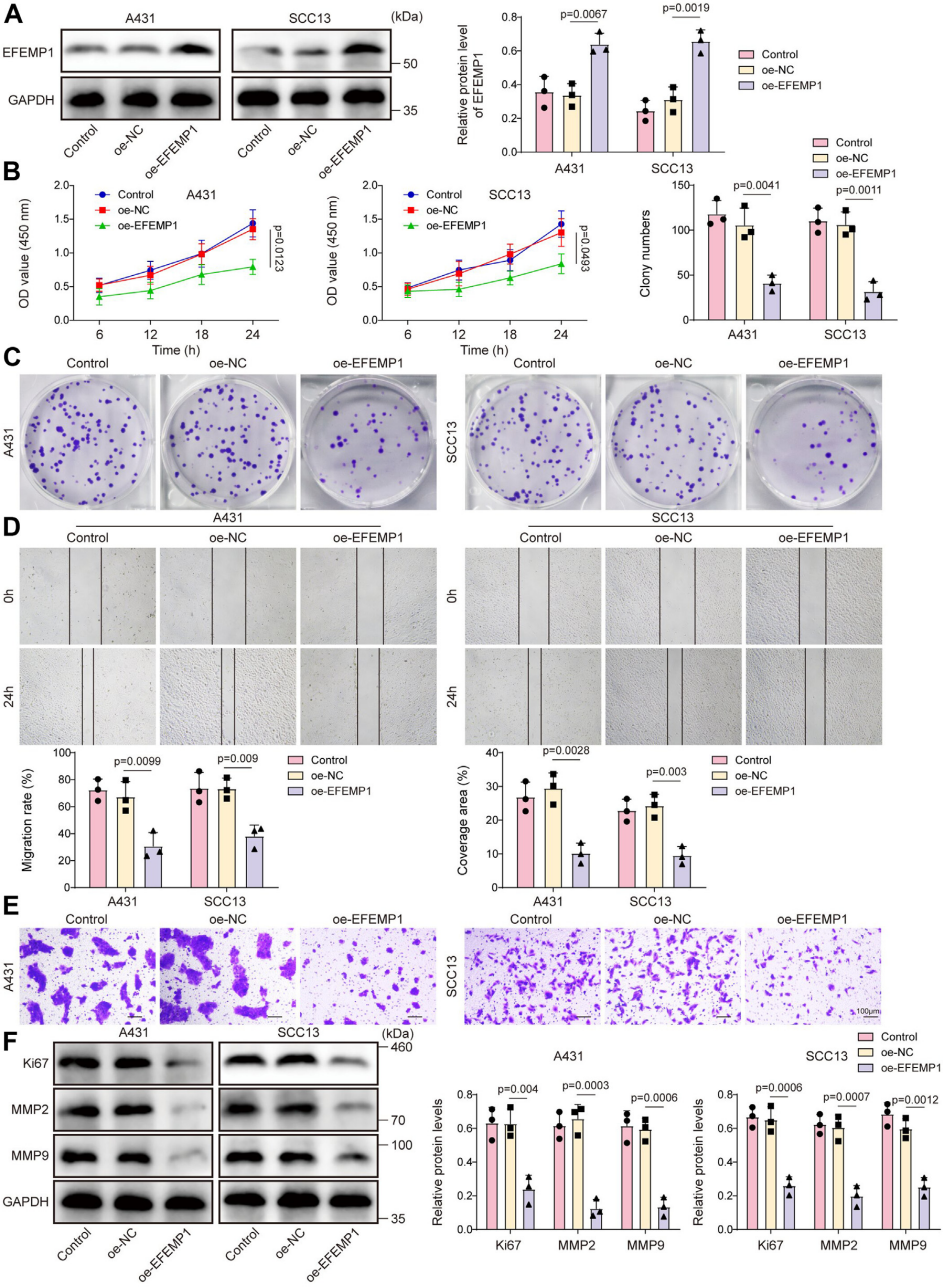
**EFEMP1 overexpression inhibited CSCC cell malignant behaviors.** A431 and SCC13 cells were transfected with oe-NC or oe-EFEMP1. After 48 h of transfection, cells were collected for further analysis. *A*, EFEMP1 protein level in cells was examined using Western blot; *p* < 0.01. *B*, cell viability was assessed using CCK8 assay; *p* < 0.05. *C*, colony formation assay was performed to examine cell proliferation; *p* < 0.01. *D*, cell migration was determined using the wound healing assay; *p* < 0.01. *E*, transwell assay was employed to analyze cell invasion (Scale bar = 100 μm); *p* < 0.01. *F*, Ki67, MMP2, and MMP9 protein levels in cells were assessed using Western blot; *p* < 0.01. One-way ANOVA was performed to compare differences between groups. All data were obtained from at least three replicate experiments and were presented as mean ± standard deviation (SD).

To study the role of EFEMP1 in regulating CSCC malignant progression, EFEMP1 overexpression was induced in CSCC cells by transfecting oe-EFEMP1 into cells. Considering that the changes of HIPK4, TAp63 phosphorylation, and EFEMP1 were more significant in A431 and SCC13 cells among all CSCC cell lines, these two CSCC cell lines were chosen for the following investigations. The transfection efficiency of oe-EFEMP1 was shown in Figure 2*A*, and the results showed that oe-EFEMP1 transfection markedly elevated EFEMP1 expression in A431 and SCC13 cells, suggesting that the transfection of oe-EFEMP1 was successful. The results subsequently revealed that EFEMP1 overexpression markedly inhibited A431 and SCC13 cell viability (Fig. 2*B*), proliferation (Fig. 2*C*), migration (Fig. 2*D*), and invasion (Fig. 2*E*). Moreover, the levels of Ki67 (proliferative marker) and epithelial–mesenchymal transformation-related proteins (MMP2 and MMP9) in A431 and SCC13 cells were significantly inhibited by EFEMP1 overexpression (Fig. 2*F*). In conclusion, EFEMP1 overexpression markedly inhibited CSCC cell malignant behaviors.

### HIPK4 knockdown inhibited CSCC cell malignant behaviors

**Figure 3:**
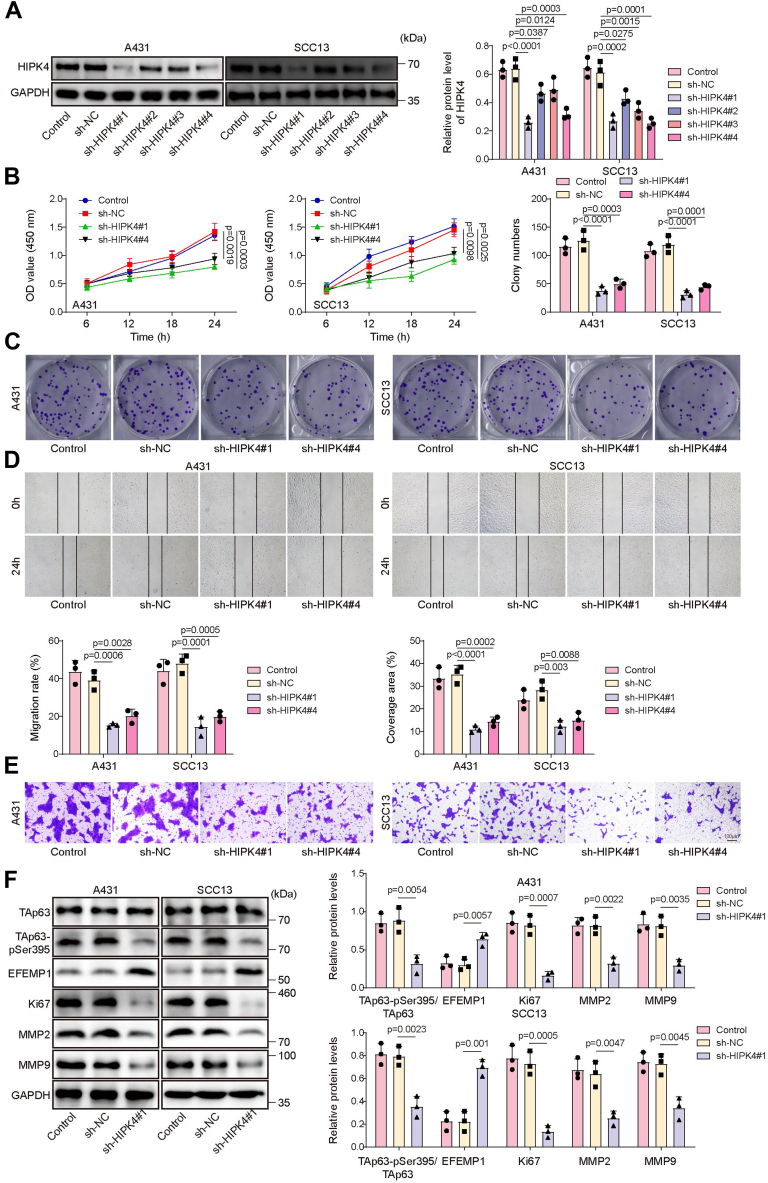
**HIPK4 knockdown inhibited CSCC cell malignant behaviors.***A*, Western blot was employed to determine HIPK4 protein level in A431 and SCC13 cells after sh-NC, sh-HIPK4#1, sh-HIPK4#2, sh-HIPK4#3, and sh-HIPK4#4 transfection; *p* < 0.05. A431 and SCC13 cells were transfected with sh-NC, sh-HIPK4#1, or sh-HIPK4#4. *B*, cell viability was examined by CCK8 assay; *p* < 0.01. *C*, colony formation assay was employed to examine cell proliferation; *p* < 0.001. *D*, cell migration was detected by wound healing assay; *p* < 0.01. *E*, transwell assay was employed to analyze cell invasion (scale bar = 100 μm); *p* < 0.01. *F*, TAp63, TAp63-pSer395, EFEMP1, Ki67, MMP2, and MMP9 protein levels in cells were assessed using western blot; *p* < 0.01. One-way ANOVA was performed to compare differences between groups. All data were obtained from at least three replicate experiments and were presented as mean ± standard deviation (SD).

To investigate the effect of HIPK4 in regulating CSCC cell malignant behaviors, HIPK4 knockdown was induced in CSCC cells by transfecting sh-HIPK4 into cells. The transfection efficiency of sh-HIPK4 is shown in Figure 3*A*, and the results showed that the transfection of sh-HIPK4#1, sh-HIPK4#2, sh-HIPK4#3, and sh-HIPK4#4 could significantly reduce HIPK4 protein level in A431 and SCC13 cells. We also observed that the knockdown efficiency of sh-HIPK4#1 and sh-HIPK4#4 was the highest among these four shRNAs; therefore, sh-HIPK4#1 and sh-HIPK4#4 were selected for subsequent experiments (Fig. 3*A*). Functional experiments subsequently showed that the CSCC cell viability (Fig. 3*B*), proliferation (Fig. 3*C*), migration (Fig. 3*D*), and invasion (Fig. 3*E*) were remarkably repressed following transfection with sh-HIPK4#1 or sh-HIPK4#4. In addition, HIPK4 knockdown significantly decreased TAp63-pSer395, Ki67, MMP2, and MMP9 levels in CSCC cells, while EFEMP1 increased (Fig. 3*F*). Taken together, CSCC cell malignant behaviors were markedly suppressed by HIPK4 knockdown.

### HIPK4 inhibited EFEMP1 expression in CSCC cells by phosphorylating TAp63 at Ser395

**Figure 4:**
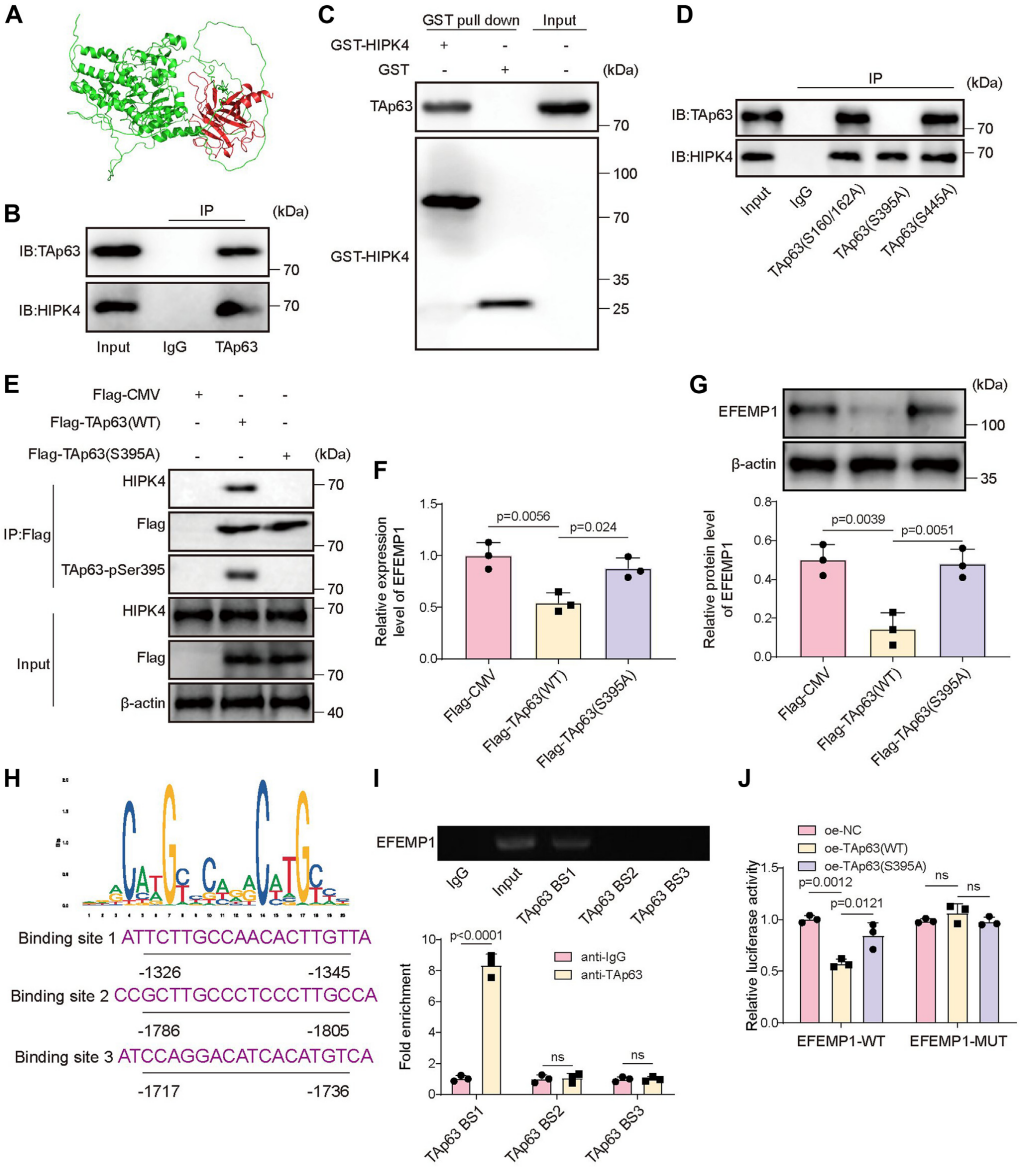
**HIPK4 inhibited EFEMP1 expression in CSCC cells by phosphorylating TAp63 at Ser395.***A*, the interaction between HIPK4 and TAp63 protein was predicted using the String database. *B* and *C*, the interaction between HIPK4 and TAp63 was analyzed by Co-IP and GST-pull down assays. *D*, different phosphorylation sites of TAp63 were mutated, and the interaction between HIPK4 and TAp63 was analyzed by Co-IP assay. *E*, A431 cells were transfected with Flag-CMV, Flag-TAp63 (WT), or Flag-TAp63 (S395A), and the binding relationship among HIPK4, TAp63, and TAp63-pSer395 was analyzed by Co-IP assay. *F* and *G*, A431 cells were transfected with Flag-CMV, Flag-TAp63 (WT), or Flag-TAp63 (S395A), and the mRNA and protein levels of EFEMP1 were analyzed by RT-qPCR and Western blot. *H*, the potential binding site between TAp63 and *EFEMP1* promoter was predicted using the JASPAR database. *I*, the interaction between TAp63 and *EFEMP1* promoter was analyzed by ChIP assay; *p* < 0.001. An aliquot of the sonicated chromatin was kept as input before precipitation. *J*, dual-luciferase reporter assays were performed to determine the relative luciferase activity in oe-NC, oe-TAp63(WT), oe-TAp63(S395A) with EFEMP1-WT or EFEMP1-MUT reporter plasmids; *p* < 0.05. One-way ANOVA was performed to compare differences between groups. All data were obtained from at least three replicate experiments and were presented as mean ± standard deviation (SD).

**Figure 5:**
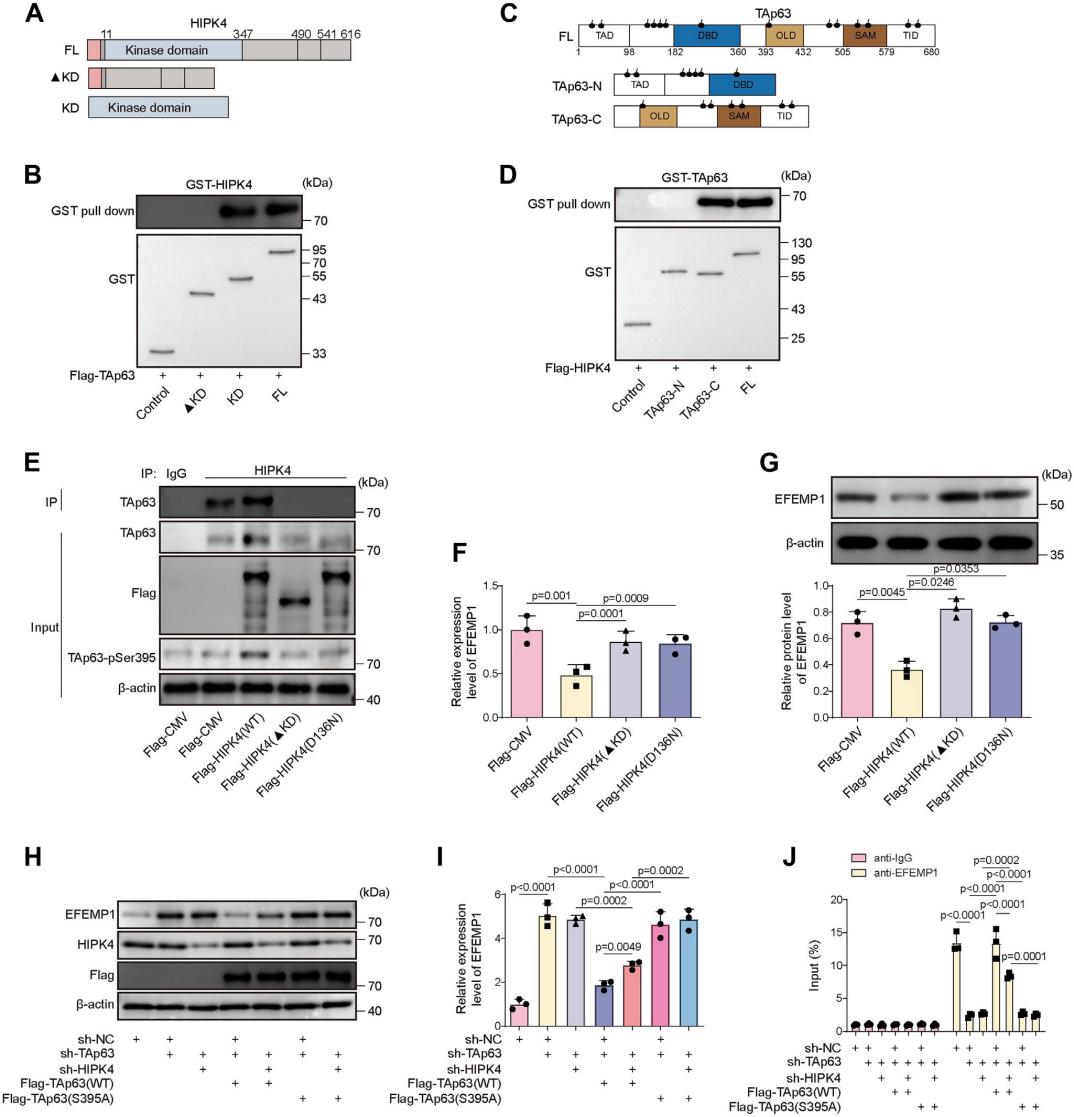
**Phosphorylation of S395 on TAp63 by HIPK4 facilitates the interaction between HIPK4 with TAp63 and subsequently inhibits the transcription and expression of EEMP1.***A*, construction of different HIPK4 truncations to identify the domain responsible for binding to TAp63. *B*, GST-pulldown assay was used to detect the interaction between GST-HIPK4 truncations and TAp63 in 293T cells. *C*, Construction of different TAp63 truncations. The truncations were designed to examine the specific binding region of TAp63 that interacts with HIPK4. *D*, GST-pulldown assay was performed to examine the interaction between GST-TAp63 truncations and HIPK4 in 293T cells. *E*, Co-IP was performed to assess the interaction between HIPK4 and TAp63 as well as the level of TAp63-pS395 in cells after the transfection of Flag-CMV, Flag-HIPK4 (WT), Flag- HIPK4 (▲KD), or Flag- HIPK4 (H136D). *F*, RT-qPCR was performed to examine the expression of EFEMP1 in cells after the transfection of Flag-HIPK4 (WT), Flag- HIPK4 (▲KD), or Flag- HIPK4 (H136D). *p* < 0.01. *G*, Western blot was performed to examine the expression of *EFEMP1* in cells after the transfection of Flag-HIPK4 (WT), Flag-HIPK4 (▲KD), or Flag- HIPK4 (H136D). *p* < 0.05. A431 cells were transfected with sh-TAp63, Flag-TAp63 (WT), Flag-TAp63 (S395A), and/or sh-HIPK4. *H*, the protein levels of EFEMP1, HIPK4, and Flag in cells were determined by western blot. *I*, the mRNA level of *EFEMP1* was determined by RT-qPCR. *p* < 0.01. *J*, the binding relationship between TAp63 and *EFEMP1* promoter was determined by ChIP. *p* < 0.001. One-way ANOVA was performed to compare differences between groups. All data were obtained from at least three replicate experiments and were presented as mean ± standard deviation (SD).

HIPK4 achieves its role in diseases by phosphorylating specific proteins (14). Herein, we predicted that HIPK4 potentially interacted with TAp63 using the molecular docking (Fig. 4*A*). Selecting A431 cells as the research object of the next experiment in this part, the Co-IP experiment demonstrated that HIPK4 was directly connected to TAp63 (Fig. 4*B*). Furthermore, GST pull-down study revealed that purified recombinant HIPK4 could pull down TAp63 (Fig. 4*C*). Subsequently, we mutated different phosphorylation sites of TAp63, and we observed that only the TAp63(S395A) could not bind to HIPK4 (Fig. 4*D*), suggesting that HIPK4 phosphorylated TAp63 at Ser395. We further verified that HIPK4 promoted phosphorylation TAp63 at Ser395 site and bound to it. As shown in Figure 4*E*, when Flag-TAp63 (WT) was transfected into cells, a strong interaction between Flag-TAp63 and HIPK4 was observed, along with an increase in TAp63-pS395 level, indicating that HIPK4 bound to phosphorylated TAp63. However, when Flag-TAp63 (S395A), a mutant with a non-phosphorylatable Ser395, was transfected, interaction between Flag-TAp63 and HIPK4, and the levels of TAp63-pSer395 were absent (Fig. 4*E*). This confirmed that the phosphorylation of TAp63 at Ser395 was crucial for the binding of HIPK4 to TAp63. Moreover, Flag-TAp63 (WT) transfection significantly increased the mRNA and protein levels of EFEMP1 in cells, but Flag-TAp63 (S395A) transfection had no significant effect on EFEMP1 expression (Fig. 4, *F* and *G*). All these results indicated that HIPK4 regulated TAp63 transcriptional activity by promoting Ser395 phosphorylation and binding to TAp63. In addition, from the prediction by using the JASPAR database, TAp63 had a potential binding site to *EFEMP1* promoter (Fig. 4*H*). In addition, this study tested the specific sites of TAp63 binding to the *EFEMP1* promoter. As confirmed by ChIP assay, the TAp63 antibody significantly enriched the fragment of *EFEMP1* binding sits 1 (EFEMP1-BS1) compared with the IgG antibody, while the TAp63 antibody didn’t enrich the fragment of *EFEMP1*-BS2 and *EFEMP1*-BS3, indicating that TAp63 could only directly bound to the promoter of EFEMP1 on the binding sits 1 (Fig. 4*I*). Dual-luciferase reporter assays were performed to determine the relative luciferase activity in oe-NC, oe-TAp63(WT), oe-TAp63(S395A) with EFEMP1-WT, or EFEMP1-MUT reporter plasmids. As shown in Figure 4*J*, significant decrease in relative luciferase activity was noted when pmiRGLO/EFEMP1-WT was co-transfected with oe-TAp63(WT) compared with the oe-NC and oe-TAp63(S395A) groups, while the relative luciferase activity in oe-TAp63(WT) group with pmiRGLO/EFEMP1-MUT reporter plasmid had no significant difference with the oe-NC and oe-TAp63-(S395A) groups with pmiRGLO/EFEMP1-MUT. Moreover, different HIPK4 truncations were constructed to investigate the functional domains of HIPK4 (Fig. 5*A*). These truncations were designed to test whether the kinase domain (KD) of HIPK4 interacts with TAp63. GST-pulldown assay was performed to test the interaction between various truncations of GST-HIPK4 and TAp63 in 293T cells. The results revealed that the KD region of HIPK4 bound directly to TAp63, as indicated by the successful pull-down of TAp63 by GST-HIPK4 truncations containing the KD domain (Fig. 5*B*). Different TAp63 truncations were created to identify the specific region of TAp63 that interacts with HIPK4, with a focus on the C-terminal region that contains Ser395 (Fig. 5*C*). As shown in Figure 5*D*, the C-terminal of TAp63 interacts with HIPK4, further confirming that HIPK4’s KD region binds to the C-terminal of TAp63, specifically implicating the Ser395 phosphorylation site in this interaction. Co-IP results also showed that Flag-HIPK4(WT) transfection significantly increased the binding between Flag-HIPK4 (WT) and TAp63 and TAp63-pS395 level (Fig. 5*E*). However, when Flag-HIPK4(▲KD), a kinase-dead mutant, was transfected, the interaction between HIPK4 and TAp63 was completely absent, and the TAp63-pS395 level decreased. Furthermore, the transfection of Flag-HIPK4(H136D), a catalytic mutant, also resulted in a reduced binding between HIPK4 and TAp63, with a corresponding decrease in TAp63-pS395 levels (Fig. 5*E*). Additionally, transfection of Flag-HIPK4(WT) led to a significant decrease in EFEMP1 mRNA and protein levels. In contrast, the transfection of Flag-HIPK4(▲KD) or Flag-HIPK4(H136D) did not affect the expression of EFEMP1, indicating that the kinase domain and the catalytic activity of HIPK4 is essential for the downregulation of EFEMP1 (Fig. 5, *F* and *G*). It also turned out that the knockdown of TAp63 resulted in a significant increase in both EFEMP1 mRNA and protein levels and a significant decrease in the binding of TAp63 to the *EFEMP1* promoter (Fig. 5, *H*–*J*). Interestingly, when HIPK4 was also knocked down in TAp63-depleted cells, no further changes were observed in EFEMP1 expression or TAp63 binding to the *EFEMP1* promoter (Fig. 5, *H*–*J*). However, when Flag-TAp63 (WT) was transfected into TAp63-knockdown cells, both EFEMP1 mRNA and protein levels were partially restored to baseline levels, and TAp63 binding to the *EFEMP1* promoter was significantly recovered (Fig. 5, *H*–*J*). Furthermore, co-transfection of Flag-TAp63 (WT) and sh-HIPK4 in TAp63 knockdown cells led to an increase in the reduced EFEMP1 expression, and a decrease in TAp63 binding to the *EFEMP1* promoter (Fig. 5, *H*–*J*). In contrast, the transfection of Flag-TAp63 (S395A) did not affect the changes induced by TAp63 knockdown (Fig. 5, *H*–*J*). Additionally, further knockdown of HIPK4 in this condition had no significant impact on EFEMP1 expression or TAp63 binding to the *EFEMP1* promoter (Fig. 5, *H*–*J*). In summary, HIPK4 phosphorylating TAp63 at Ser395, and inhibited EFEMP1 expression in CSCC cells.

### HIPK4 promoted CSCC malignant progression by inhibiting EFEMP1 expression through phosphorylating TAp63

To verify whether HIPK4 promotes the malignant progression of CCSC cells by regulating the TAp63/EFEMP1 axis, HIPK4 knockdown and/or EFEMP1 knockdown were induced in CSCC cells.

**Figure 6:**
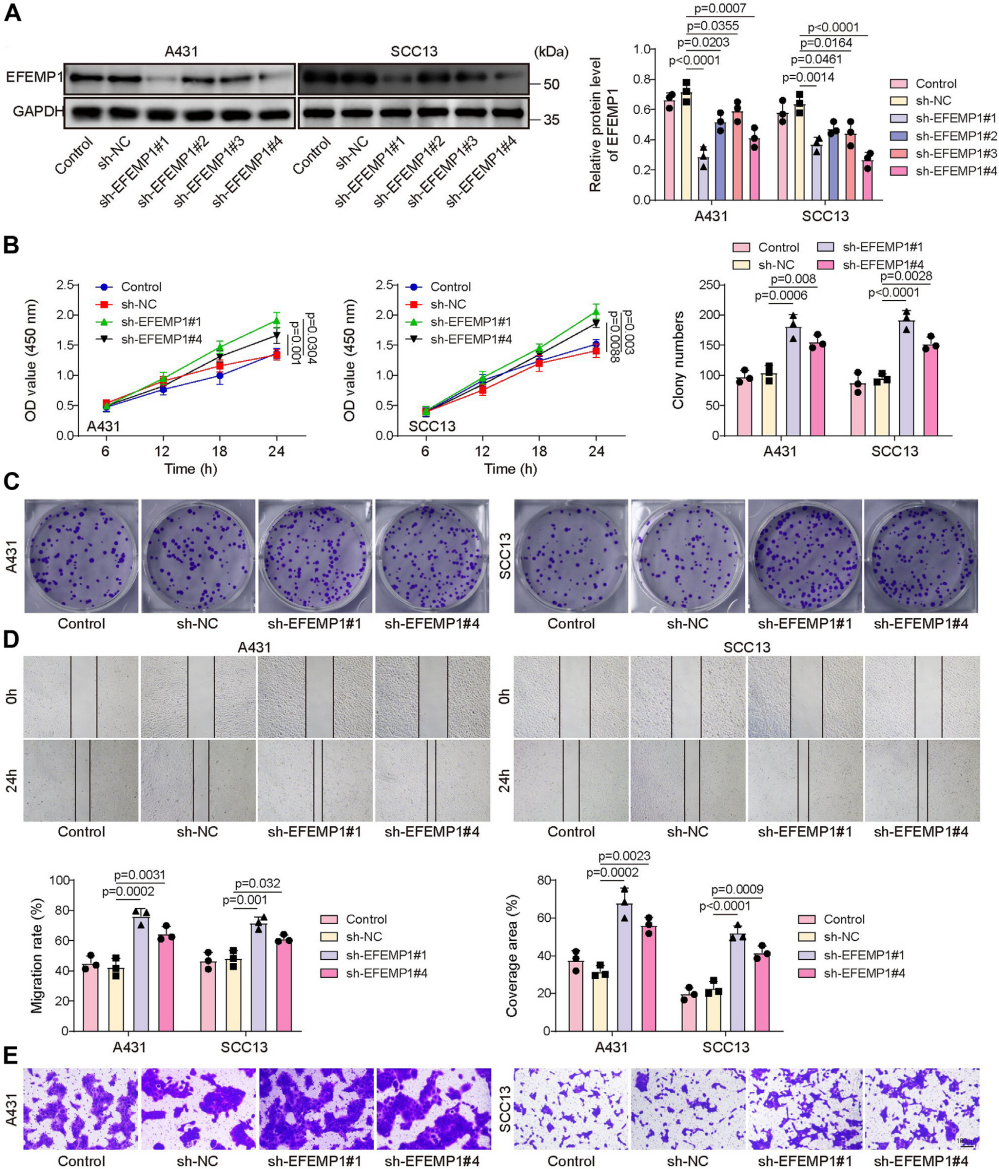
**EFEMP1 knockdown facilitated CSCC cell malignant behaviors.***A*, Western blot was employed to determine EFEMP1 protein level in A431 and SCC13 cells after sh-NC, sh-EFEMP1#1, sh-EFEMP1#2, sh-EFEMP1#3, or sh-EFEMP1#4 transfection; *p* < 0.05. A431 and SCC13 cells were transfected with sh-NC, sh- EFEMP1#1, or sh-EFEMP1#4. *B*, cell viability was examined by CCK8 assay; *p* < 0.05. *C*, colony formation assay was employed to examine cell proliferation; *p* < 0.01. *D*, cell migration was detected by wound healing assay; *p* < 0.05. *E*, transwell assay was employed to analyze cell invasion (scale bar = 100 μm); *p* < 0.01. The measurement data were presented as mean ± SD. One-way ANOVA was performed to compare differences between groups. All data were obtained from at least three replicate experiments.

The transfection efficiency of sh-EFEMP1 was shown in Figure 6*A*, and the results showed that both the transfection of sh-EFEMP1#1, sh-EFEMP1#2, sh-EFEMP1#3, and sh-EFEMP1#4 could significantly reduce EFEMP1 protein level in A431 and SCC13 cells. We also observed that the knockdown efficiency of sh-EFEMP1#1 and sh-EFEMP1#4 was the highest among these four shRNAs; therefore, sh-EFEMP1#1 and sh-EFEMP1#4 were selected for subsequent experiments (Fig. 6*A*). As shown in Figure 6, *B*–*E*, the transfection of sh-EFEMP1#1 and sh-EFEMP1#4 significantly promoted CSCC cell viability, proliferation, migration, and invasion, but the transfection of sh-EFEMP1#1 showed more significant effects compared to sh-EFEMP1#4. Therefore, sh-EFEMP1#1 was selected for subsequent experiments. In addition, HIPK4 silencing reduced HIPK4 and TAp63-pSer395 levels and elevated EFEMP1 level in A431 and SCC13 cells, while EFEMP1 knockdown reversed the promoting effect of HIPK4 silencing on EFEMP1 expression but did not affect HIPK4 and TAp63-pS395 protein levels significantly (Fig. S1*A*). Additionally, EFEMP1 knockdown significantly prevented HIPK4 downregulation-induced decrease in CSCC cell viability (Fig. S1*B*), proliferation (Fig. S1*C*), migration (Fig. S1*D*), and invasion (Fig. S1*E*). Moreover, HIPK4 knockdown exerted an inhibitory effect on Ki67, MMP2, and MMP9 expression in CSCC cells, which was relieved after sh-EFEMP1 transfection (Fig. S1*F*). Moreover, HIPK4 knockdown significantly reduced HIPK4 and TAp63-pS395 levels but increased EFEMP1 level in A431 cells, but the co-transfection of Flag-TAp63 (WT) significantly reduced EFEMP1 level, and Flag-TAp63-S395A co-transfection did not affect the changes caused by HIPK4 knockdown (Fig. S2*A*). In addition, the co-transfection of Flag-TAp63 (WT) reversed the inhibitory effects of HIPK4 silencing on CSCC cell viability, proliferation, migration, and invasion, but Flag-TAp63 (S395A) co-transfection did not affect the changes caused by HIPK4 knockdown (Fig. S2, *B*–*E*). In conclusion, HIPK4 promoted CSCC cell malignant behaviors by decreasing EFEMP1 expression through phosphorylating TAp63 at Ser395.

### HIPK4 knockdown inhibited tumor growth in mice

**Figure 7:**
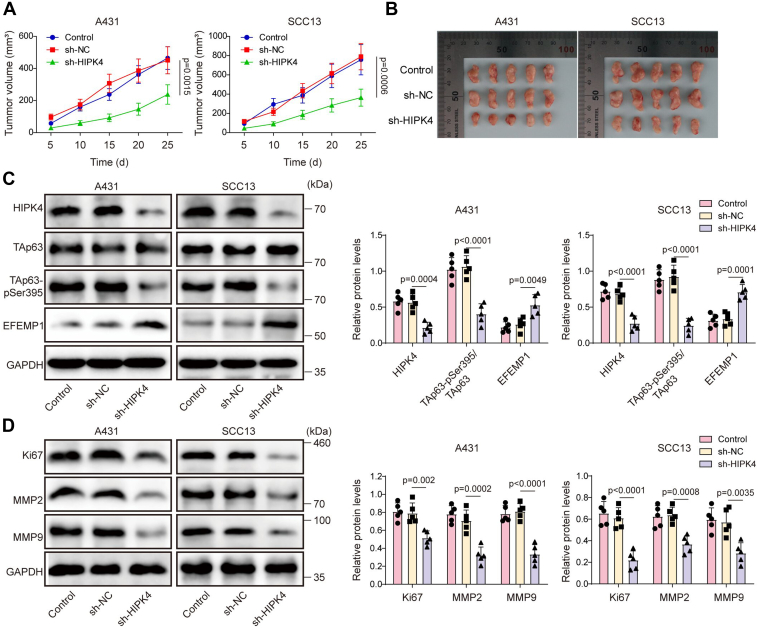
**HIPK4 knockdown reduced tumor growth in mice.** The tumor implantation experiment was performed in BALB/c nude mice (4-5 weeks old, 18-20 g), which were randomly divided into two groups: control (sh-NC) and HIPK4 knockdown (sh-HIPK4). A total of 2 × 10^6^ A431 cells stably transfected with sh-NC or sh-HIPK4 were subcutaneously injected into the right flank of each mouse. The tumor volume was measured every 5 d. The mice were then euthanized 25 d after injection, and the tumor tissues were collected for further analysis. (*A* and *B*) The size and volume of tumors were measured; *p* < 0.01. (*C* and *D*) HIPK4, TAp63, TAp63-pSer395, EFEMP1, Ki67, MMP2, and MMP9 protein levels in tumor tissues were examined using western blot; *p* < 0.01. n=5, One-way ANOVA was performed to compare differences between groups and was presented as mean ± standard deviation (SD).

To further study the effect of HIPK4 on CSCC malignant progression *in vivo*, we established a subcutaneous tumor model in nude mice. Compared with that in the control group, the tumor weight and volume were significantly reduced in the HIPK4-knockdown group (Fig. 7, *A* and *B*). Western blot results subsequently displayed that HIPK4 knockdown significantly reduced HIPK4, TAp63-pS395, Ki67, MMP2, and MMP9 levels, but elevated EFEMP1 levels in tumor tissues (Fig. 7, *C* and *D*). To sum up, HIPK4 silencing inhibited tumor growth *in vivo* by regulating the TAp63/EFEMP1 axis.

## Discussion

CSCC progression occurs rapidly, with 5% of cases with CSCC having lymph node metastasis, which is the fundamental cause of non-melanoma cancer mortality (21). A recent study showed that 62.6% of CSCC patients had lymph node metastasis, of which 20.2% developed distant metastasis, showing that treating metastatic CSCC is a substantial problem (22). Therefore, further research should be conducted on the molecular mechanisms underlying the progression of CSCC to identify new diagnostic and therapeutic targets and improve treatment outcomes. Our current findings showed that HIPK4 promoted CSCC malignant progression by inhibiting EFEMP1 through phosphorylating TAp63.

EFEMP1 is an extracellular matrix glycoprotein (23) that participates in complicated biological processes such as cell proliferation and migration (24). As reported, EFFMP1 has both pro-tumor and anti-tumor properties (25). As evidence, EFEMP1 upregulation was associated with a poor outcome in ovarian carcinoma (26). In addition, EFEMP1 overexpression promoted osteosarcoma cell migration and invasion (24). However, an opposite effect of EFEMP1 in cancer has also been observed. Hu *et al.* revealed that EFEMP1 upregulation could inhibit malignant glioma growth (27). The particular method of molecular control of EFEMP1 in various malignancies is unclear and appears to need additional exploration. Our results displayed that EFEMP1 was lowly expressed in CSCC tissues and cells. Consistent with our study, a previous study showed that EFEMP1 was reduced in CSCC tissues, and its overexpression inhibited CSCC tumor growth (20). MMPs are a class of proteases that play a key role in the degradation of the extracellular matrix. As representative members, MMP2 and MMP9 are essential for physiological processes such as cell migration and invasion (28, 29). As previously reported (30), EFEMP1 reduced the expression of MMP2 and MMP9 by regulating the activity of ERK1/2, thereby inhibiting the migration of cancer cells. Our results also showed that EFEMP1 overexpression markedly inhibited CSCC cell proliferation, migration, and invasion. Collectively, EFEMP1 upregulation inhibited CSCC malignant progression.

The *HIPK4* gene is located in the region of chromosome 19q13.2 and encodes a conserved serine/threonine kinase protein (31). HIPK4 can regulate skin development and early epithelial differentiation through various signaling pathways (13). However, the role of HIPK4 in CSCC hasn’t been reported before. Our results showed that HIPK4 was highly expressed in CSCC, and its silencing inhibited CSCC cell malignant behaviors and tumor growth in CSCC-bearing nude mice, which was reported for the first time.

HIPK4 regulation has not been extensively studied to date. As a conserved serine/threonine kinase, HIPK4 achieves its function by phosphorylating proteins (14). As revealed by Arial *et al.*, HIPK4 phosphorylates human p53 at Ser9 (15). A similar mechanism was observed in the current study. Our findings demonstrated that HIPK4 phosphorylated TAp63 at Ser395. TAp63 is a subtype of p63, which belongs to the p53 transcription factor family. (9). As reported, TA isoforms tend to have tumor suppressor activities (32). As expected, the role of TAp63 as a tumor suppressor and invasion/metastasis inhibitor has been extensively explored in the past few years. As evidence, Lin *et al.* revealed that TAp63 overexpression suppressed colon cancer cell migration and invasion (33). Importantly, TAp63 could inhibit CSCC progression by suppressing a network of cell-cycle genes (11). TA isoforms retain clear transcriptional regulatory functions, which can bind DNA and promote transcription (32). Herein, our findings demonstrated that TAp63 transcriptionally inhibited EFEMP1 expression by binding to the *EFEMP1* promoter. As expected, EFEMP1 downregulation ameliorated sh-HIPK4-induced decrease in CSCC cell malignant behaviors. All these results suggested that HIPK4 promoted CSCC malignant progression by phosphorylating TAp63 and inhibiting EFEMP1.

In the current study, we failed to clarify the upstream regulation mechanism of HIPK4 in CSCC development, which is a limitation of our study. We will continue to explore the upstream regulatory mechanism of HIPK4, as conditions permit in the future, to make our research more comprehensive. In addition, HIPK4 may also have other related targets other than TAp63 in CSCC, which are involved in the progression of CSCC through multiple pathways and mechanisms, and its role may be more complex than currently believed. Therefore, in addition to continuing to explore the upstream regulatory mechanism of HIPK4, identifying other potential targets of HIPK4 in CSCC is also important for a more comprehensive understanding of its role in the disease. Taken together, HIPK4 promoted CSCC malignant progression by inhibiting TAp63-mediated EFEMP1 transcription activation through phosphorylating TAp63 at Ser395. As a result, HIPK4 may serve as a biological marker or target for the diagnosis or therapy of CSCC. In future investigations, we will look into HIPK4's clinical relevance as a diagnostic and prognostic marker for CSCC.

## Experimental procedures

### Clinical sample collection

CSCC tumor tissues and adjacent tissues were collected from patients with CSCC at the First Affiliated Hospital of Anhui Medical University. All patients with CSCC were diagnosed through pathological examination, including histopathological assessment of biopsy specimens to confirm the diagnosis and accurately classify the tissues as cancerous or adjacent non-cancerous. Tumor and normal tissue samples were obtained during surgery, ensuring proper sample handling and minimizing potential degradation. The study adhered to the principles of the Declaration of Helsinki, guaranteeing ethical standards for human subject research. The Clinical Research Ethics Committee of the First Affiliated Hospital of Anhui Medical University approved this study (ethical number PJ2024–05–44), and each patient signed an informed consent form, indicating their understanding of the study purpose and procedures. Patients were provided with detailed information regarding the use of their tissue samples for research purposes, and all steps followed stringent ethical guidelines for informed consent and patient confidentiality. All tissue samples were immediately snap-frozen and stored at −80 °C for long-term preservation and future molecular analysis, ensuring the integrity and quality of the specimens for further genetic and biochemical investigations.

### Cell culture

The human immortalized epidermal cells (HaCaT cells) and human CSCC cell lines (A431 and MET-2 cells) were provided by the Shanghai Institute of Biological Sciences. SCL-1 cells were obtained from Jennio Biotech. The SCC13 cells were provided by the Chinese Academy of Medical Sciences, and the HSC-5 cells were sourced from Sekisui Xenotech. 293T cells were purchased from American Type Culture Collection (ATCC). All cell lines were cultured in RPMI 1640 medium (Gibco) supplemented with 10% fetal bovine serum (FBS; Gibco) and incubated in a humidified atmosphere with 5% CO_2_ at 37 °C to maintain optimal growth conditions. The cells were passaged regularly when they reached 70 to 80% confluence to avoid overcrowding and ensure consistent growth. Prior to experimental use, all cell lines were authenticated using short tandem repeat (STR) analysis to confirm the correct identity of the cell lines and avoid cross-contamination. Additionally, cell cultures were regularly monitored for *mycoplasma* contamination. Mycoplasmas were detected using both the arginine broth culture method and the semi-fluid culture method, which are well-established techniques for identifying *mycoplasma* contamination in cell culture supernatants. These precautions were taken to ensure the integrity of the cells and the accuracy of the experimental results.

### Cell transfection

The short hairpin RNAs (sh-HIPK4 and sh-EFEMP1), the overexpression plasmid (oe-EFEMP1 and oe-TAp63), and their negative controls were purchased from GenePharma. We have designed three shRNAs for HIPK4 and EFEMP1 respectively (sh-HIPK4#1, sh-HIPK4#2, sh-HIPK4#3, and sh-HIPK4#4; sh-EFEMP1#1, sh-EFEMP1#2, sh-EFEMP1#3, and sh-EFEMP1#4, sequences were showed in Table S1). The vectors and shRNAs were introduced into cells using Lipofectamine 3000 (Invitrogen). To achieve efficient transfection, the vectors and shRNAs were introduced into cells using Lipofectamine 3000 (Invitrogen) according to the manufacturer’s instructions. Briefly, for transfection, cells were seeded at a density of 60 to 70% confluence in a 6-well plate and allowed to adhere for 24 h. The required amount of plasmid DNA or shRNA (1–2 μg for each well) was mixed with Lipofectamine 3000 reagent (1:1 ratio of DNA to Lipofectamine) in opti-MEM medium (Gibco) and incubated for 20 to 30 min at room temperature to allow complex formation. The DNA-Lipofectamine complexes were then added to the cells, and transfection was carried out for 48 h before analysis. Control cells were transfected with the respective negative control vectors or shRNAs. After transfection, cells were harvested for further experiments, including Western blot analysis, RT-qPCR, and functional assays.

### Animal experiments

SJA LABORATORY provided 30 male BALB/c nude mice (4-5-week-old; 18–20 g). Mice were housed under pathogen-free conditions with a 12-h light/dark cycle and maintained at a constant temperature of 22 ± 2 °C and humidity of 50 to 60%. All mice had ad libitum access to food and acidified water. Mice were randomly classified into three groups: control, sh-NC and sh-HIPK4 (with five mice in each group). 0.1 ml PBS containing 2 × 10^6^ A431 or SCC13 cells which stable transfected with adenovirus-carrying sh-NC or sh-HIPK4 were subcutaneously inoculated in the axilla of nude mice. The tumor volume was measured every 5 days. The mice were then euthanized 25 days after injection, and the tumor tissues were collected. The size was measured (V = half × the length of the tumor × width^2^). All animal experiments were operated in accordance with the “Laboratory Animal Management Treaty” and were approved by the Anhui Medical University and the ethical number is LLSC20241764.

### Cell counting kit-8 (CCK-8) assay

A431 and SCC13 cells were collected and resuspended in culture medium to obtain a cell density of 5 × 10^5^ cells per mL. Cell suspensions were then added to a 96-well plate, with each well containing 100 μl of cell suspension (5 × 10ˆ4 cells per well). The plate was incubated at 37 °C with 5% CO_2_ for 24 h to allow the cells to adhere. After 24 h, 10 μl of CCK-8 solution (Yeason) was added to each well. The cells were then incubated for an additional 1.5 h at 37 °C in a CO_2_ incubator. Following the incubation, the absorbance was measured at 450 nm using a microplate reader (Thermo Fisher Scientific). The absorbance values were used to calculate cell viability. Each experiment was performed in triplicate, and cell viability was calculated as a percentage relative to the control group.

### Colony formation assay

A431 and SCC13 cells were seeded into 6-well plates at a density of 1 × 10^3^ cells per well. The cells were cultured for 7 days in complete culture medium (DMEM supplemented with 10% FBS, 100 U/ml penicillin, and 100 μg/ml streptomycin) at 37 °C with 5% CO_2_. The medium was replaced every 2 to 3 days. After 7 days, the colonies were fixed with 4% paraformaldehyde (Sigma-Aldrich) for 30 min at room temperature. Following fixation, the colonies were stained with 0.1% (w/v) crystal violet solution (Sigma-Aldrich) for 10 min at room temperature. The stained colonies were washed with PBS to remove excess dye and then air-dried. The colonies were counted under a microscope, and the number of colonies with more than 50 cells was recorded. The colony formation efficiency was calculated as the ratio of the number of colonies to the number of seeded cells.

### Wound healing assay

A431 and SCC13 cells were seeded in 6-well plates at a density of 5 × 10^5^ cells per well and cultured in complete DMEM medium (with 10% FBS, 100 U/ml penicillin, and 100 μg/ml streptomycin) for 12 h to allow for cell attachment. After 12 h, the culture medium was removed, and a uniform scratch wound was created using a sterile 200 μl pipette tip. The cells were then washed twice with PBS to remove any debris. Fresh DMEM without serum was added to the wells to prevent further cell proliferation. Images of the scratch were captured using a light microscope at 0 and 24 h after scratching to observe cell migration and wound closure. The wound area was quantified using ImageJ software by measuring the area of the wound at both time points. The relative wound closure percentage was calculated by comparing the wound areas at 0 and 24 h.

### Transwell assay

A total of 1 × 10^4^ cells resuspended in 500 μl serum-free DMEM were added to the upper chamber of a Transwell insert pre-coated with 100 μl of Matrigel for the invasion assay. In the bottom chamber, 1000 μl of complete DMEM (containing 10% FBS) was added to act as a chemoattractant. The cells were allowed to invade through the membrane for 12 h at 37 °C with 5% CO_2_. After the incubation period, the culture medium was removed from both chambers. Cells that did not invade, located on the upper side of the membrane, were gently wiped off with a cotton swab. The remaining cells that adhered to the underside of the membrane were fixed with 4% paraformaldehyde (Sigma-Aldrich) for 30 min at room temperature. Following fixation, the cells were stained with 0.1% crystal violet solution (Sigma-Aldrich) for 10 min to visualize the invaded cells. After staining, the membrane was rinsed with PBS to remove excess dye. The cells were then observed under a light microscope (Olympus) at 200 × magnification, and the number of cells that had invaded was counted in five random fields. The data were presented as the average number of cells per field.

### RT-qPCR

Total RNA was extracted from cells using the RNAiso Plus kit (Takara Bio) according to the manufacturer's instructions. The RNA was quantified using a NanoDrop spectrophotometer (Thermo Fisher Scientific), and cDNA was synthesized using the PrimeScript RT Reagent Kit (Takara Bio). RT-qPCR was performed using SYBR Premix Ex Taq (Takara Bio) with specific primers for *EFEMP1* and *GAPDH*. The relative expression of *EFEMP1* was calculated using the 2^∧^(−ΔΔCt) method. The following primers were used:

*EFEMP1* (F): 5′-GCCAGAGACCTGAGGGGAG-3′

*EFEMP1* (R): 5′-GGAGAGAGGGCAGGAAGAA-3′

*GAPDH* (F): 5′-TGGGAGGAGTGGGACG-3′

*GAPDH* (R): 5′-TGGGAGGAGTGGGACG-3′

### Western blot analysis

Total protein extraction was performed using RIPA lysis buffer (Beyotime) containing a protease inhibitor cocktail (Roche) to prevent protein degradation. Protein concentrations were determined using a BCA Protein Assay Kit (Thermo Fisher Scientific). A total of 20 μg of protein was loaded onto a 10% SDS-PAGE gel and subjected to electrophoresis at 120V for 90 min to separate the proteins based on size. After electrophoresis, the proteins were transferred onto a PVDF membrane (Millipore) using a semi-dry transfer method at 100V for 1 h. The membrane was then blocked with 5% non-fat milk (Bio-Rad) in Tris-buffered saline with 0.1% Tween-20 (TBST) for 1 h at room temperature to prevent non-specific binding. The membranes were then incubated overnight with antibodies against HIPK4 (Abcam, ab69565, 1:1000), TAp63 (Abcam, ab124762, 1:1000), TAp63-pSer395 (Abcam, ab92581, 1:1000), EFEMP1 (Abcam, ab151976, 1:2000), Ki67 (Abcam, ab16667, 1:1000), matrix metalloproteinase (MMP)2 (Abcam, ab16667, 1:1000), MMP9 (Abcam, ab76003, 1:5000), Flag (Abcam, ab236777, 1:5000), β-actin (Abcam, ab8226, 1:5000) and GAPDH (Abcam, ab8245, 1:5000). After overnight incubation, the membranes were washed three times with TBST for 5 min each to remove any unbound primary antibody. The membranes were then incubated with the secondary antibody (Abcam, ab7090,1:5000) for 60 min. After washing three times with TBST, the protein bands were visualized using an enhanced chemiluminescence (ECL) substrate (Beyotime). The chemiluminescent signal was detected using a chemiluminescence imaging system (Bio-Rad,). The intensity of the protein bands was quantified using ImageJ software (NIH), and the data were normalized to GAPDH or β-actin levels as an internal loading control.

### Dual luciferase reporter assay

The fragment of the *EFEMP1* promoter containing the TAp63 binding site was amplified by PCR using specific primers. The primers were designed to include restriction sites compatible with the pmiRGLO vector (Promega). Site-directed mutagenesis of the TAp63 binding site within the *EFEMP1* promoter fragment was performed using a Site-Directed Mutagenesis Kit (Stratagene) following the manufacturer’s instructions to generate the mutated (MUT) reporter plasmid. The wild-type (WT) and mutated (MUT) *EFEMP1* promoter sequences were cloned into the pmiRGLO vector, which contains both firefly luciferase and Renilla luciferase reporter genes. Cells were co-transfected with 1 μg of the WT or MUT EFEMP1 reporter plasmids along with 1 μg of overexpression plasmids for wild type TAp63 (oe-TAp63(WT)) TAp63 with a Ser395 mutation (oe-TAp63(S395A)), or the negative control (oe-NC) using Lipofectamine 3000 reagent (Invitrogen) according to the manufacturer’s protocol. The transfection medium was replaced with fresh DMEM 6 h after transfection, and cells were incubated for an additional 48 h. Luciferase activity was then measured using the Dual-Luciferase Reporter Assay System (Promega) according to the manufacturer’s instructions. Firefly luciferase activity was measured first, followed by the measurement of Renilla luciferase activity. The ratio of Renilla to firefly luciferase activity was used to normalize the results, allowing for accurate comparison of luciferase activity between different groups.

### Chromatin immunoprecipitation (ChIP)

ChIP assay was performed using the EZ-Magna Chip A/G Chromatin Immunoprecipitation Kit (Millipore) according to the manufacturer's protocol. In brief, A431 cells were cultured to 70 to 80% confluence and then treated with 1% formaldehyde (Sigma-Aldrich) for 5 min at room temperature to cross-link proteins to DNA. The reaction was quenched by adding glycine to a final concentration of 0.125 M, and cells were washed with cold PBS. The cell pellets were collected, resuspended in cell lysis buffer provided in the kit, and incubated on ice for 10 min. Chromatin was fragmented by ultrasonication using a Bioruptor Plus (Diagenode) at high intensity for 10 cycles (30 s ON, 30 s OFF). The sonicated chromatin was centrifuged at 12,000 rpm for 10 min at 4 °C to remove cell debris, and the supernatant containing chromatin fragments was collected. To immunoprecipitate specific protein-DNA complexes, the chromatin was incubated overnight at 4 °C with primary antibodies against TAp63 (Abcam, 1:50, ab124762) or control IgG (Abcam, 1:100, ab172730). Protein A/G magnetic beads (Pierce, Thermo Fisher Scientific) were added to the chromatin-antibody mixture and incubated for 1 h at 4 °C with rotation to allow the chromatin-antibody complexes to bind. The complexes were washed with low-salt, high-salt, and LiCl wash buffers provided in the kit to remove non-specific binding. Chromatin-bound proteins were eluted from the beads by adding elution buffer and heating at 65 °C for 15 min. Cross-links were reversed by incubating the samples overnight at 65 °C. The eluted DNA was purified using the Spin Column provided in the kit. The purified DNA was analyzed by agarose gel electrophoresis, and the DNA fragments were visualized under UV light. An aliquot of the sonicated chromatin before immunoprecipitation was kept as the input control for normalization.

#### Protein structure modeling

To predict the binding properties of HIPK4 on TAp63, we used PDB file for HIPK4 and TAp63 from AlphaFold Protein Structure Database (https://alphafold.ebi.ac.uk/). The binding pose of HIPK4 in the binding site of TAp63 was predicted by GRAMM server (https://gramm.compbio.ku.edu/). Then, the structures of the HIPK4 and TAp63 protein were presented by PyMOLsoftware (version 3.0.5).

### Coimmunoprecipitation (Co-IP)

Cells were transfected with Flag-CMV, Flag-TAp63 (WT), or TAp63(S395A). In the another group, cells were transfected with Flag-CMV, Flag-HIPK4 (WT), Flag- HIPK4 (▲KD), or Flag-HIPK4 (H136D). Flag-CMV, Flag-TAp63 (WT), TAp63(S395A), Flag-HIPK4 (WT), Flag- HIPK4(▲KD), and Flag-HIPK4(H136D) were constructed by Genepharma. Cells were then lysed in lysis solution including protease inhibitors. The cell lysates were incubated overnight with sepharose CL-4B beads (Sigma-Aldrich) pre-bound to IgG (Abcam, 1:50, ab172730), TAp63 (Abcam, 1:50, ab124762), Flag (Abcam, 1:50, ab255980), and HIPK4 (Abcam, 1:50, ab69565) antibodies. TAp63(S395A) was constructed by mutating serine to alanine A in the Ser395 site sequence TSIKKRRsPDDELLY of TAp63. TAp63(S160/162A) was constructed by mutating serine at the SSTFDALsPsPAIPSNT sequence site to alanine A. TAP63(S445A) was constructed by mutating serine at the QKQtIQsPSSYGNs sequence site to alanine A. Then the bound proteins were eluted and used for Western blot analysis.

### GST-pull down

Bacteria expressing GST, GST-HIPK4, GST-HIPK4 truncations, or GST-TAp63 truncations were coupled to glutathione-Sepharose 4B beads (GE Healthcare) and incubated with 293T cells for 2 h at 4 °C. The complexes were then washed with GST-binding buffer to remove unbound proteins. After washing, the complexes were eluted with SDS-PAGE loading buffer by boiling. The interaction between GST-HIPK4 truncations and TAp63 was analyzed by western blotting. To investigate the binding specificity, GST-pulldown assays were performed with different truncations of both HIPK4 and TAp63, and the results were visualized using anti-TAp63 or HIPK4 antibodies.

### Data analysis

All data were obtained from three independent trials. The statistical data were analyzed using GraphPad Prism 7.0 and expressed as mean ± standard deviation (SD). The differences between the two groups were investigated using Student’s *t* tests. One-way ANOVA was performed to compare differences between groups. The *p* values less than 0.05 were regarded as significant.

## Data availability

All data generated or analyzed during this study are included in this published article.

## Supporting information

This article contains supporting information. The content that HIPK4 promoted CSCC malignant progression by inhibiting EFEMP1 expression through phosphorylating TAp63 was provided in Figure S1 and the content that HIPK4 phosphorylates TAp63 at S395, thereby promoting cell proliferation, migration and invasion was provided in Figure S2. The shRNA targeting sequences were listed as in Table S1.

## Ethics approval and consent to participate

The Clinical Research Ethics Committee of The First Affliated Hospital of Anhui Medical University approved this study, the ethical number is PJ2024-05 to 44, and each patient signed an informed consent form.

The animal studies were approved by the Anhui Medical University and the ethical number is LLSC20241764.

## Consent for publication

Informed consent was obtained from study participants.

## Conflict of interest

The authors declare that they have no conflicts of interest with the contents of this article.
