# Supplementary material for: HIPK4 accelerates cutaneous squamous cell carcinoma progression by phosphorylating TAp63 and inhibiting EFEMP1 expression
Source: J Biol Chem. 2025 Apr 30;301(7):108564. doi: 10.1016/j.jbc.2025.108564 (PMC12284529; doi:10.1016/j.jbc.2025.108564)
Supplement: Supporting information [file mmc1.pdf]

Supplementary Materials

1. Supplementary Figure and Figure legends

Figure S1. HIPK4 promoted CSCC malignant progression by inhibiting EFEMP1 expression through phosphorylating TAp63.

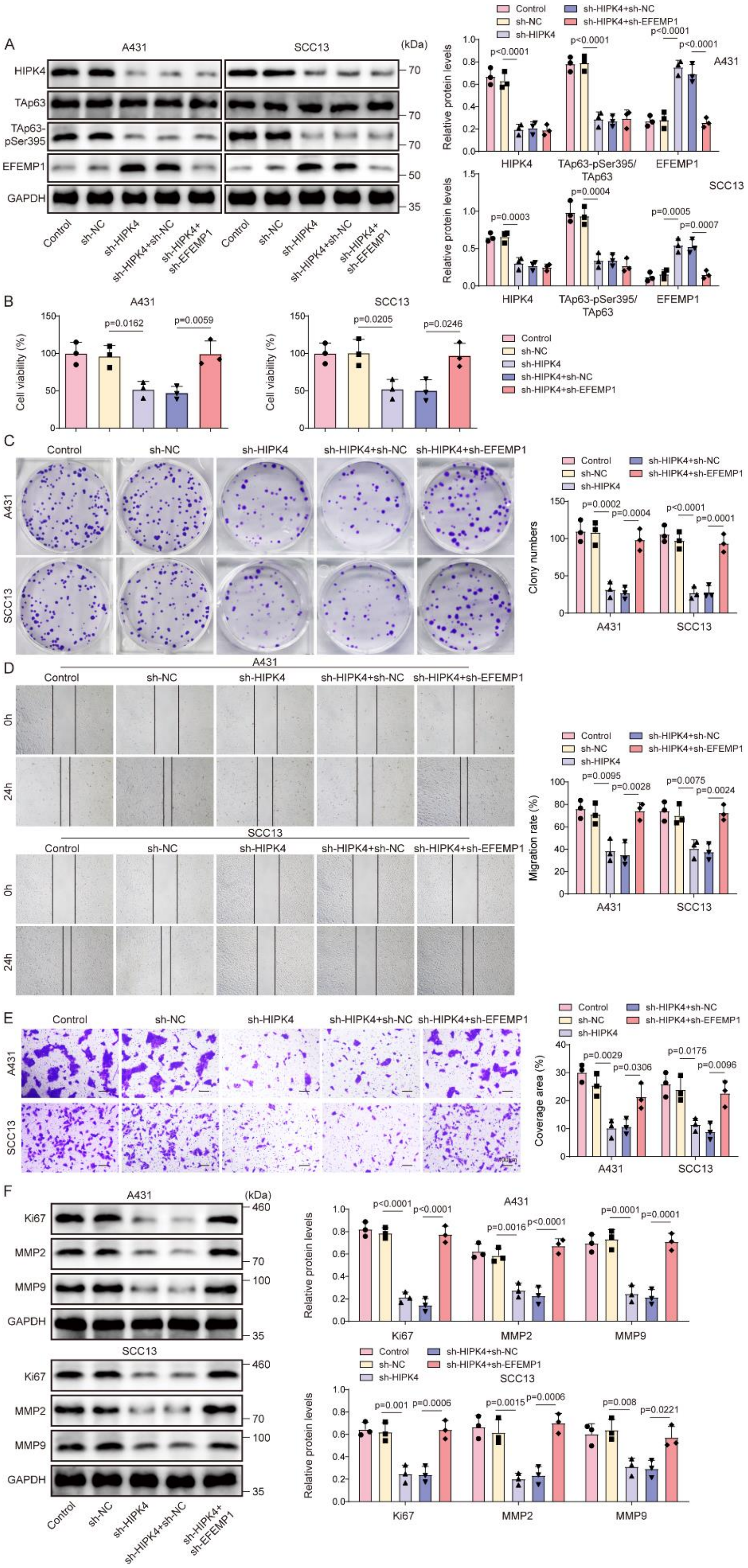

A431 and SCC13 cells were transfected with sh-NC, sh-HIPK4, sh-EFEMP1, or co-

transfected with sh-HIPK4 and sh-EFEMP1. After 48 h of transfection, cells were collected for further analysis. (A) HIPK4, TAp63, TAp63-pSer395, and EFEMP1 protein levels in cells were examined using western blot; $p < 0.001$. (B) Cell viability was examined by CCK8 assay; $p < 0.05$. (C) Colony formation assay was employed to examine cell proliferation; $p < 0.001$. (D) Cell migration was detected by wound healing assay; $p < 0.01$. (E) Transwell assay was employed to analyze cell invasion (Scale bar = 100 μm); $p < 0.05$. (F) Ki67, MMP2, and MMP9 protein levels in cells were assessed using western blot; $p < 0.05$. One-way ANOVA was performed to compare differences between groups. All data was obtained from at least three replicate experiments, and was presented as mean ± standard deviation (SD).

Figure S2. HIPK4 phosphorylates TAp63 at S395, thereby promoting cell proliferation, migration and invasion.

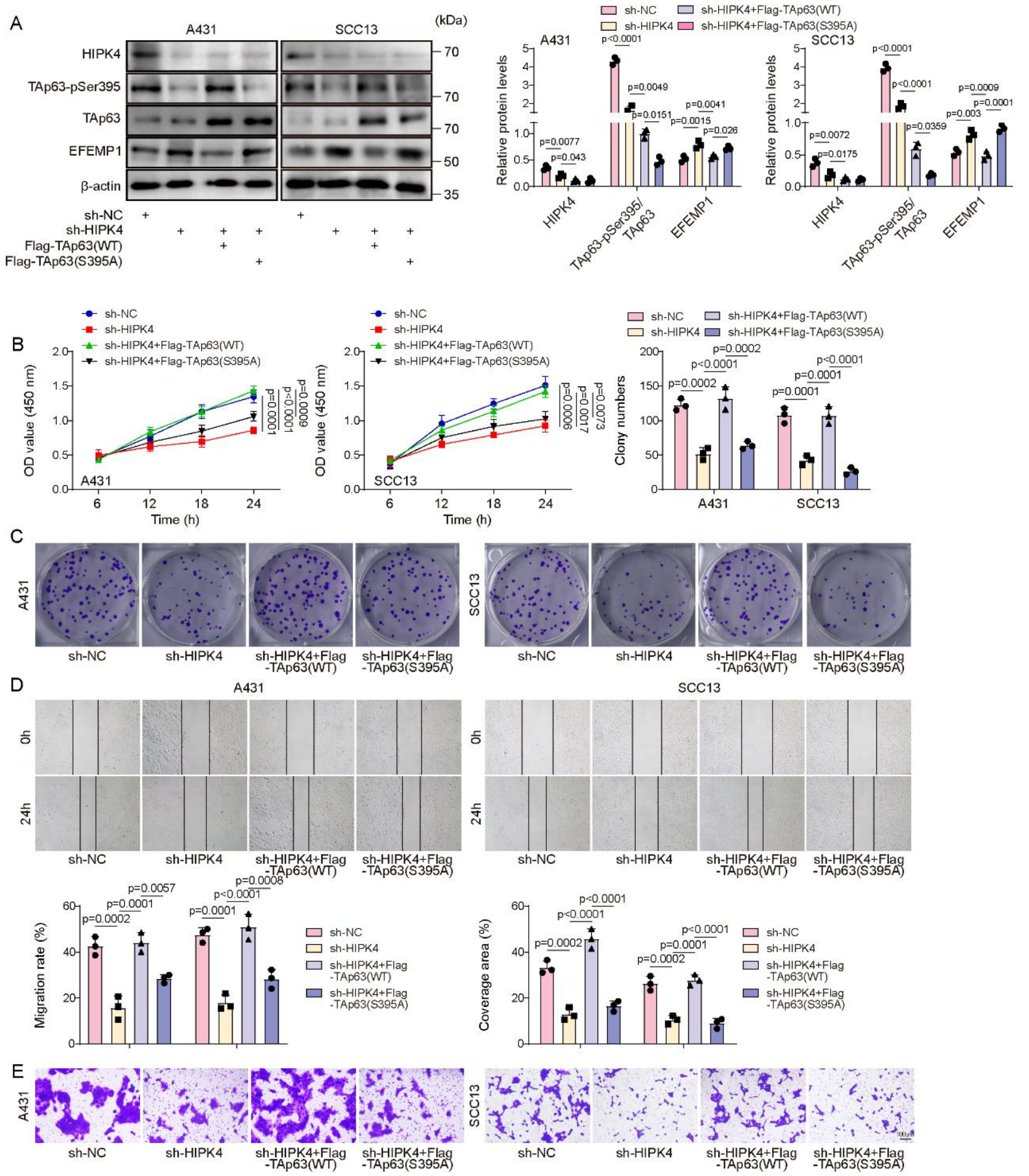

A431 cells were transfected with shNC, shHIPK4, Flag-TAp63 (WT), and/or Flag-Tap63 (S395A). (A) Western blot was employed to detect the protein levels of HIPK4, TAp63-pS395, TAp63, and EFEMP1 in cells. (B) Cell viability was examined by CCK8 assay; $p < 0.05$. (C) Colony formation assay was employed to examine cell proliferation; $p < 0.001$. (D) Cell migration was detected by wound healing assay; $p < 0.01$. (E) Transwell assay was employed to analyze cell invasion (Scale bar = 100 μm); $p < 0.001$. One-way ANOVA was performed to compare differences between groups. All data was

obtained from at least three replicate experiments, and was presented as mean ± standard deviation (SD).

2. Supplementary Table and table legends

Table S1 The shRNA targeting sequences were listed as follows:

Name	shRNA targeting sequences
shHIPK4-1#	5’-CGCTTCCTTGAGTTCTTCCAT-3’
shHIPK4-2#	5’-GCCGCAAGTATATGCTCAAGT-3’
shHIPK4-3#	5’-CTACGTGAAGGAGCCATACAT-3’
shHIPK4-4#	5’-GCTCAAGTCCTCGGCTGACTA-3’
shEFEMP1-1#	5’-GCCCAGATTATTGTCAATAAT-3’
shEFEMP1-2#	5’-GCCACCAAAGATGCGTGAATA-3’
shEFEMP1-3#	5’-GCGTAGACATAGATGAATGTA-3’
shEFEMP1-4#	5’-CCAGTCAATAGTCTACAAATA-3’
